# Work–family balance and work engagement as predictors of turnover among newly hired blue-collar workers in the automotive supplier industry: a 6-month prospective study

**DOI:** 10.3389/fpubh.2026.1841023

**Published:** 2026-07-15

**Authors:** Ezgi Sarıkız, Nergis Kayacan Başer

**Affiliations:** Department of Occupational Health and Safety, Institute of Graduate Education, Bandirma Onyedi Eylul Universitesi, Bandırma, Türkiye

**Keywords:** automotive industry, blue-collar workers, employee turnover, work engagement, work–family balance

## Abstract

**Objective:**

This prospective cohort study examined whether work–family balance and work engagement were associated with employer-verified turnover over a 6-month follow-up among newly hired blue-collar workers in the automotive supplier industry, within the framework of the Job Demands–Resources model.

**Methods:**

The study was conducted in an automotive supplier company in the Bandırma Organized Industrial Zone, Türkiye. A total of 334 blue-collar workers with a job tenure of 0–6 months were included. Data were collected using a sociodemographic questionnaire, a work–family balance scale, and the short form of the Utrecht Work Engagement Scale. Participants were followed for 6 months, and turnover status was verified using company records. Descriptive statistics, group comparisons, chi-square tests, Spearman correlation analyses, and parsimonious adjusted binary logistic regression were performed to examine the factors associated with turnover.

**Results:**

The median age was 26.5 years, and 76.6% of participants were women. At the end of follow-up, 16.2% of workers had left their jobs. Workers who left had higher scores on the “negative impact of work on family life” dimension, whereas work engagement scores were higher among those who remained employed. Job choice motivation was also associated with turnover, with higher turnover observed among those who reported negative reasons for choosing the job. Work engagement was negatively correlated with work–family conflict dimensions and positively correlated with family–work harmony. In the parsimonious adjusted logistic regression model, work engagement was significantly associated with turnover status. Higher work engagement was associated with lower odds of turnover (OR = 0.948; 95% CI: 0.902–0.996), whereas the negative effect of work on family, negative job preference group, and baseline tenure were not statistically significant after adjustment.

**Conclusion:**

Higher work engagement was associated with lower early turnover among newly hired blue-collar workers in the automotive supplier industry. Although work–family balance dimensions were related to psychosocial functioning and showed univariate differences, they did not remain statistically significant in the adjusted parsimonious model. Workplace strategies that strengthen engagement and support work–family balance may contribute to improved workforce stability in manufacturing settings.

## Introduction

The automotive industry is a strategic manufacturing sector characterized by highly intensive work processes operating under stringent quality standards, tight delivery deadlines, and the pressure of just-in-time production (1). In this context, the sector’s intensive production structure may impose a substantial psychosocial burden on blue-collar workers, extending beyond physical fatigue (1). In a global competitive environment, the ability of enterprises to sustain productivity and quality depends not only on the optimization of technical processes but also on the management of organizational and psychosocial factors that shape employees’ work experiences and retention behaviors (2, 3).

In manufacturing organizations, personnel mobility, including turnover and department or line changes, may have important consequences for organizational sustainability. Employee turnover is associated not only with recruitment, replacement, and training costs, but also with productivity losses, disruption of work routines, loss of organizational knowledge, and reduced operational continuity (4–7). Empirical studies have shown that turnover and turnover volatility may adversely affect labor productivity, while the costs of turnover may include both direct replacement expenses and indirect costs related to short staffing, onboarding, and team performance (4–7). Indeed, meta-analytic findings have shown that high labor turnover rates are consistently and strongly negatively associated with indicators of organizational performance (2, 3). In this context, interventions aimed at increasing personnel stability are of vital importance not only for human resource management but also for overall quality, continuity, and cost management.

The Job Demands–Resources model provides a theoretically robust framework for explaining workforce turnover and its underlying mechanisms. The JD-R approach proposes that every job has its own specific demands, such as time pressure and shift work, as well as resources, such as autonomy and social support (8, 9). This framework provides a useful basis for understanding how job demands, job resources, work engagement, and organizational outcomes may be related, without implying that a formal mediation pathway is tested in the present study. Work engagement refers to a positive, energetic psychological state toward one’s work, encompassing the dimensions of vigor, dedication, and absorption (10). Particularly among newly hired blue-collar employees, examining factors that are associated with work engagement and early turnover may help clarify psychosocial conditions relevant to workforce stability.

Within the JD-R framework, the work–family interface can be conceptualized as a multidimensional construct. Conflict-related dimensions, such as the negative effect of work on family and the negative effect of family on work, may operate as demand-related components, whereas harmony-related dimensions may function as resources that support wellbeing and work engagement. More than simply the absence of role conflict, work–family balance reflects the individual’s perception of being able to manage work and family roles in a harmonious and satisfying way (11, 12). Considering the demanding shift systems and time pressure in manufacturing enterprises, unfavorable work–family experiences may be associated with reduced work engagement and a higher risk of personnel mobility.

The aim of this prospective follow-up study was to examine whether perceptions of work–family life balance and levels of work engagement were associated with employer-verified turnover after 6 months of follow-up among newly hired blue-collar workers with 0–6 months of tenure in an automotive supplier company located in northwestern Türkiye. Based on the JD-R model, we hypothesized that more favorable work–family balance perceptions and higher work engagement would be associated with a lower likelihood of actual turnover during the early employment period. The study did not aim to test a formal mediation pathway; instead, it examined the direct associations of work–family balance dimensions and work engagement with actual turnover. The primary contribution of this study is its focus on objectively verified turnover behavior rather than the more commonly used self-reported measure of turnover intention. By linking early psychosocial perceptions with a concrete employment outcome, the study provides evidence that may inform workplace interventions aimed at reducing early workforce loss in manufacturing settings.

## Materials and methods

### Study design and theoretical framework

This study is a prospective, observational, and quantitative investigation examining the relationship between work-family life balance, work engagement, and personnel mobility at the end of the follow-up period among newly hired blue-collar workers in the automotive supplier industry. The theoretical foundation of the study was built on the Job Demands-Resources model. Within this framework, the work–family interface was operationalized as a multidimensional construct including both demand-related conflict dimensions and a harmony-related resource dimension, work engagement as an indicator of the motivational process, and actual turnover behavior as an organizational outcome.

### Study population and participants

The study was conducted in a large-scale supplier company producing for the global automotive industry in a major organized industrial zone located in northwestern Türkiye. Production processes in the company are carried out in a shift-based system under strict performance targets. The target population consisted of all blue-collar employees working on site and directly involved in production line activities who had a job tenure of 0–6 months at the time of baseline data collection. This tenure criterion was selected because the organizational adaptation process is considered more fragile among newly hired employees, and psychosocial perceptions such as work–family balance and work engagement may develop rapidly during this early employment period.

No sampling procedure was applied. Instead, a census approach was used, and all eligible workers who met the inclusion criterion during the data collection period were invited to participate. A total of 334 eligible employees voluntarily agreed to participate and were included in the study. Since there were no missing or erroneous data, all 334 questionnaires were included in the final analysis.

### Data collection instruments

Research data were collected through a structured questionnaire consisting of a form assessing participants’ sociodemographic and occupational characteristics and two standardized scales:

- *Sociodemographic and occupational information form*: This form, developed by the researchers based on a literature review, consisted of 19 questions covering work-related variables such as sex, age, marital status, having children, educational level, and shift pattern.
- *Work-Family Life Balance Scale*: The Work–Family Life Balance Scale developed by Apaydın was used to measure employees’ perceptions of balance between work and family roles (13). The scale consists of 12 items rated on a five-point Likert scale and includes three subdimensions: Negative Effect of Work on Family, Negative Effect of Family on Work, and Family-Work Harmony. Higher scores indicate higher levels of perception in the relevant dimension. For this study, the overall internal consistency coefficient of the scale was found to be highly reliable (Cronbach’s alpha = 0.84).
- *Utrecht Work Engagement Scale*: Work engagement was assessed using the six-item Turkish version of the Utrecht Work Engagement Scale. The original UWES framework and its short form were developed by Schaufeli et al. (14), while the Turkish validity and reliability of the short version was examined by Özkalp and Meydan (15). In addition, the six-item Turkish version of the scale was proposed by Güler et al. (16) as an alternative version for assessing the three dimensions of work engagement, namely vigor, dedication, and absorption. The scale was rated on a five-point Likert scale, and a higher total score indicated a higher level of work engagement. In the present study, the Cronbach’s alpha coefficient of the scale indicated excellent reliability (*α* = 0.91).

### Data collection process and follow-up protocol

The purpose, scope, and voluntary nature of the study were explained in detail to the participants, and written informed consent was obtained. Objective questions related to demographic and occupational characteristics were administered by the researchers through face-to-face interviews, whereas the scale items measuring psychosocial perceptions were completed by the participants themselves in order to minimize social desirability bias. Baseline data collection was completed within a two-week period.

Following the initial data collection phase, all participants were prospectively followed for 6 months. At the end of the follow-up period, the current employment status of the workers in the company (still employed or left the job) was verified through the official Human Resources records of the institution and matched with the baseline survey data through unique codes assigned to the participants. Through this follow-up protocol, the study was able to objectively measure actual turnover behavior verified by institutional records, rather than relying on turnover intention, which is more commonly used in the literature.

### Ethical approval and data security

This study was conducted in strict accordance with the principles of the Declaration of Helsinki. Official approval for the study protocol was obtained from the Non-Interventional Clinical Research Ethics Committee of a state university (Decision No: 2025-1, Date: 29.01.2025). In addition, written institutional permission was obtained from the management of the industrial company where the data were collected. No identifying information, such as names or employee identification numbers, was included on the consent forms or questionnaires; data matching was performed using a coded system known only to the researchers. Physical documents were stored in locked areas, and digital data were secured on encrypted devices accessible only to the research team.

### Statistical analysis

Data analysis was performed using IBM SPSS Statistics version 21.0. Before analysis, the dataset was examined for outliers, coding errors, and assumptions of normality. Statistical significance was accepted as *p* < 0.05.

Descriptive statistics were reported as frequency and percentage for categorical variables and as mean ± standard deviation or median (interquartile range) for continuous variables, depending on data distribution. Since continuous variables did not show normal distribution according to the Shapiro–Wilk test (*p* < 0.05), non-parametric methods were preferred for hypothesis testing. The Mann–Whitney *U* test was used for comparisons between two independent groups, and the Kruskal–Wallis test was used for comparisons among three or more groups. The source of significant differences was determined using Dunn’s *post-hoc* test with Bonferroni correction. Relationships between categorical variables were examined using Pearson’s chi-square test, and relationships between continuous or ordinal variables were analyzed using Spearman’s rank correlation analysis. Effect sizes were calculated for Mann–Whitney *U* tests using the formula *r* = |*Z*|/√*N* and were reported in the relevant comparison tables. For Spearman correlation analyses, the correlation coefficient itself was interpreted as an indicator of the direction and strength of the association.

Since turnover behavior, the main dependent variable of the study, had a dichotomous structure (0 = still employed, 1 = left employment), binary logistic regression analysis was performed to examine factors associated with turnover behavior. In response to the limited number of turnover events and to avoid overfitting, a parsimonious adjusted model was constructed. Variables were selected based on theoretical relevance and univariate findings. The final model included work engagement, the negative effect of work on family, job preference group, and baseline tenure in months. Baseline tenure was included to account for potential differences in organizational adjustment at the time of enrolment.

The results were presented as adjusted odds ratios and 95% confidence intervals. The overall fit of the logistic model was evaluated using the Omnibus Test of Model Coefficients and the Hosmer–Lemeshow goodness-of-fit test, while the explanatory power of the model was assessed using the Nagelkerke *R*^2^ statistic. Individual coefficients were interpreted within the final model only after confirming the statistical significance of the overall model.

## Results

### Descriptive characteristics of the study group

The study was completed with the participation of 334 blue-collar workers who met the specified eligibility criteria. The median age of the participants was 26.5 years (IQR: 23.0–41.0), and the majority were women (76.6%, *n* = 256). The proportions of single (48.2%) and married (44.3%) employees were similar, and 42.8% of the participants had children, most commonly two children (54.5%). In terms of educational level, most employees were high school graduates (46.1%) or university graduates (24.9%). In addition, more than half of the participants were smokers (53.0%), whereas chronic illness (8.7%) and disability (1.8%) were observed at relatively low rates (Table 1).

**Table 1:** Sociodemographic and health characteristics of the participants (*N* = 334).

Variable	Category	*n*	%
Sex	Female	256	76.6
	Male	78	23.4
Marital status	Single	161	48.2
	Married	148	44.3
	Divorced	25	7.5
Educational status	Primary school	62	18.6
	Middle school	35	10.5
	High school	154	46.1
	University	83	24.9
Having children	No	191	57.2
	Yes	143	42.8
Number of children*	1 child	45	31.5
	2 children	78	54.5
	3 children	15	10.5
	4 children	5	3.5
Smoking	Non-smoker	157	47.0
	Smoker	177	53.0
Chronic disease	No	305	91.3
	Yes	29	8.7
Disability	No	328	98.2
	Yes	6	1.8

Age (years): median 26.5; P25 = 23.0; P75 = 41.0; Min–Max = 18–58; SD = 10.886.

^*^In these rows, the denominator includes only participants with children (*n* = 143).

### Working life and employment status at follow-up

Examination of the participants’ working conditions showed that most were employed in the Trim (58.4%) and Quality (26.9%) departments and were distributed relatively evenly across the Yellow, Blue, and Green shift groups. A substantial proportion of the employees (74.6%) reported that they had chosen their current job for positive reasons, whereas approximately one-quarter (25.4%) stated that they had started the job due to negative reasons such as financial necessity, obligation, or the availability of a vacant position.

At the end of the six-month follow-up period, 83.8% of the participants were still working in the company, whereas 16.2% (*n* = 54) had left the institution. The vast majority of those who left (83.3%) had done so voluntarily, while only a small proportion (16.7%) had been dismissed by the employer. Among participants who reported considering a job change (*n* = 54), the main motivations were heavy workload or financial expectations (48.1%) and the desire to move into the field in which they had been educated (46.3%) (Table 2).

**Table 2:** Work-related characteristics and job preferences (*N* = 334).

Variable	Category	*n*	%
Employment status at follow-up	Working	280	83.8
	Left employment	54	16.2
Reason for leaving**	Voluntary resignation	45	83.3
	Dismissed	9	16.7
Department	Trim	195	58.4
	Quality	90	26.9
	Human resources	3	0.9
	Logistics	23	6.9
	Cutting	18	5.4
	Maintenance	5	1.5
Shift	Yellow	108	32.3
	Blue	123	36.8
	Green	103	30.8
Reason for job choice (group)	Negative (financial reasons/necessity/vacant position)	85	25.4
	Positive (suitable/related to my education/suitable for women)	249	74.6
Thought of changing job	No	280	83.8
	Yes	54	16.2
Reason for change***	Heavy workload, financial reasons	26	48.1
	Transfer to the department corresponding to their education	25	46.3
	Personal adjustment problems	3	5.6

^**^Denominator includes only participants who left employment (*n* = 54).

^***^Denominator includes only participants who reported considering a job change (*n* = 54).

### Comparisons according to demographic characteristics and employment status

When the relationships between categorical variables were evaluated, a statistically significant clustering was identified between department of employment and educational level (*p* < 0.001). University graduates were more commonly employed in the Quality department, whereas lower educational levels were associated with greater concentration in production areas such as Trim and Cutting.

Job-entry motivation was associated with employment status at follow-up. The rate of leaving the job was significantly higher among those who had started work for negative reasons such as financial necessity, obligation, or a vacant position (23.5%) compared with those who had started for positive reasons (13.7%) (*p* = 0.033). In contrast, no statistically significant association was found between having children and actual job leaving (*p* = 0.216) (Table 3).

**Table 3:** Distribution of participants who remained employed and those who left employment according to parental status and job preference group.

Variable		Working, *n* (%)	Left employment, *n* (%)	Total	*p*
Parental status	No	156 (81.7)	35 (18.3)	191	0.216
	Yes	124 (86.7)	19 (13.3)	143	
	Total	280 (83.8)	54 (16.2)	334	
Job preference group	Negative (financial reasons, obligation, vacant position)	65 (76.5)	20 (23.5)	85	0.033
	Positive (suitable, aligned with my education, appropriate for women)	215 (86.3)	34 (13.7)	249	
	Total	280 (83.8)	54 (16.2)	334	

### Psychosocial perceptions: work-family balance and work engagement

At follow-up, the median scores for the “negative effect of work on family” were significantly higher among employees who had left the job than among those who remained employed (*p* = 0.032). In contrast, levels of work engagement were significantly higher in the group that continued working (*p* = 0.011).

Demographic characteristics were found to differentiate psychosocial perceptions. Married participants and those with children had significantly higher work engagement scores than single participants and those without children (*p* < 0.001). Among single or divorced individuals, the “negative effect of family on work” was perceived more strongly (*p* = 0.038). In addition, among those who had chosen the job for negative reasons from the outset, both dimensions of work–family conflict were higher and work engagement remained at a lower level (*p* < 0.05) (Table 4).

**Table 4:** Comparison of Work–Family/Life Balance and Work Engagement Scale scores by sociodemographic characteristics of participants.

Group		Scale variable groups							
		Work–Family/Life Balance Scale						Work Engagement Scale (total)	
		Negative effect of work on family		Negative effect of family on work		Family–work harmony			
		Mean	SD	Mean	SD	Mean	SD	Mean	SD
Employment status	Working (*n* = 280)	11.14	4.93	2.94	1.67	14.24	4.19	22.73	5.51
	Left job (*n* = 54)	12.74	5.21	3.44	2.14	14.41	4.39	20.41	6.47
	*Z*	−2.142		−1.200		−0.379		−2.543	
	*r*	0.117		0.066		0.021		0.139	
	*p*	**0.032**		0.230		0.705		**0.011**	
Marital status	Single/divorced (*n* = 186)	11.90	5.43	3.17	1.83	13.95	4.01	21.55	5.76
	Married (*n* = 148)	10.77	4.35	2.83	1.65	14.66	4.44	23.36	5.55
	*Z*	−1.555		−2.074		−2.159		−3.769	
	*r*	0.085		0.113		0.118		0.206	
	*p*	0.120		**0.038**		**0.031**		**0.001**	
Parental status	No children (*n* = 191)	12.01	5.41	3.16	1.81	14.34	3.79	21.38	5.67
	Has children (*n* = 143)	10.59	4.30	2.84	1.67	14.17	4.74	23.64	5.57
	*Z*	−2.106		−2.045		−0.509		−4.689	
	*r*	0.115		0.112		0.028		0.257	
	*p*	**0.035**		**0.041**		0.611		**< 0.001**	
Job preference	Negative content (*n* = 85)	13.27	5.23	3.48	2.00	14.20	4.42	21.22	5.44
	Positive content (*n* = 249)	10.76	4.77	2.86	1.64	14.29	4.15	22.73	5.78
	*Z*	−3.901		−3.079		−0.142		−2.557	
	*r*	0.213		0.168		0.008		0.140	
	*p*	**<0.001**		**0.002**		0.887		**0.011**	

SD, standard deviation; Z, Mann–Whitney *U* test statistic. Bold *p* values indicate statistical significance at *p* < 0.05. Effect size r was calculated using the formula *r* = |*Z*|/√*N*.

Correlation analyses showed that work engagement was negatively associated with work–family conflict dimensions and positively associated with family–work harmony. Moderate negative correlations were found between work engagement and the “negative effect of work on family” (*r* = −0.410; *p* < 0.001) and the “negative effect of family on work” (*r* = −0.326; p < 0.001). As age increased, work engagement showed a weak positive correlation (*r* = 0.192; p < 0.001), whereas the negative effect of work on family showed a weak negative correlation with age (*r* = −0.126; *p* = 0.022) (Table 5).

**Table 5:** Spearman correlation analysis between age, work-family balance scale subdimensions, and work engagement.

Correlation method	Variables	Statistic	Age	Negative effect of work on family	Negative effect of family on work	Family–work harmony	Work engagement
Spearman’s rho	Age	*r*	1.000	−0.126^*^	−0.087	0.016	0.192^**^
		*p*		0.022	0.114	0.768	< 0.001
	Negative effect of work on family	*r*	−0.126^*^	1.000	0.497^**^	0.067	−0.410^**^
		*p*	0.022		<0.001	0.221	<0.001
	Negative effect of family on work	*r*	−0.087	0.497^**^	1.000	0.074	−0.326^**^
		*p*	0.114	<0.001		0.178	<0.001
	Family–work harmony	*r*	0.016	0.067	0.074	1.000	0.257^**^
		*p*	0.768	0.221	0.178		<0.001
	work engagement	*r*	0.192^**^	−0.410^**^	−0.326^**^	0.257^**^	1.000
		*p*	<0.001	<0.001	<0.001	<0.001	

*Correlation is significant at the 0.05 level (2-tailed).

**Correlation is significant at the 0.01 level (2-tailed).

*r*, correlation coefficient.

### Adjusted associations with turnover: parsimonious binary logistic regression analysis

A parsimonious adjusted binary logistic regression model was constructed to examine the associations of work engagement, the negative effect of work on family, job preference group, and baseline tenure with turnover status (Table 6). These variables were selected based on theoretical relevance and the univariate findings, while non-essential sociodemographic covariates were excluded from the primary model to reduce model complexity.

**Table 6:** Parsimonious adjusted binary logistic regression model for turnover status among newly hired blue-collar workers.

Variable	*B*	Standard error	*p*-value	Adjusted odds ratio	95% confidence interval
Work engagement	−0.053	0.025	0.034	0.948	0.902–0.996
Negative effect of work on family	0.034	0.031	0.262	1.035	0.975–1.099
Negative job preference group (vs positive)	0.527	0.327	0.107	1.695	0.893–3.215
Baseline tenure, months	0.039	0.101	0.699	1.040	0.853–1.267

Dependent variable: turnover status; 0 = still employed, 1 = left employment.

Reference category for job preference group: positive job preference reasons.

Model variables: work engagement, negative effect of work on family, job preference group, and baseline tenure in months.

Model fit statistics: Omnibus Test of Model Coefficients, *χ*^2^ = 11.613, df = 4, *p* = 0.020; Hosmer–Lemeshow goodness-of-fit test, *χ*^2^ = 12.095, df = 8, *p* = 0.147; Nagelkerke *R*^2^ = 0.058.

The overall model was statistically significant according to the Omnibus Test of Model Coefficients (*χ*^2^ = 11.613, df = 4, *p* = 0.020). The Hosmer–Lemeshow goodness-of-fit test indicated acceptable model fit (*χ*^2^ = 12.095, df = 8, *p* = 0.147), and the Nagelkerke *R*^2^ value was 0.058.

In this parsimonious adjusted model, work engagement was significantly associated with turnover status. Each one-unit increase in work engagement was associated with lower odds of turnover (OR = 0.948; 95% CI: 0.902–0.996; *p* = 0.034). The negative effect of work on family, negative job preference group, and baseline tenure were not statistically significant after adjustment.

These findings indicate that higher work engagement was associated with lower early turnover among newly hired blue-collar workers. Although the negative effect of work on family and negative job preference reasons showed significant univariate associations with turnover, these variables did not remain statistically significant in the adjusted parsimonious model.

## Discussion

In this prospective follow-up study of newly hired blue-collar workers with 0–6 months of tenure in the automotive supplier industry, employees who left their jobs had higher scores for the “negative effect of work on family,” whereas those who remained employed had higher work engagement scores. In the parsimonious adjusted logistic regression model, work engagement was significantly associated with lower odds of turnover. In contrast, the negative effect of work on family and negative job preference reasons, although significant in univariate analyses, did not remain statistically significant after adjustment. These findings suggest that work–family conflict may be relevant to early turnover at the descriptive and univariate level, while work engagement appears to be the psychosocial factor most consistently associated with early retention in the adjusted parsimonious model.

The finding that work–family conflict was higher among employees who left their jobs is consistent with the broader literature on the adverse consequences of conflict between work and family roles. Allen et al. (17) showed in their review that work–family conflict is associated with a wide range of adverse outcomes, including lower job satisfaction, weaker organizational attitudes, stress, and health-related problems. Amstad et al. (18) also reported in their meta-analysis that both directions of work–family conflict, namely the effect of work on family and the effect of family on work, were consistently associated with work-related, family-related, and general wellbeing outcomes. Our study supports this general framework by showing that work–family conflict dimensions are related to psychosocial functioning among newly hired blue-collar workers. However, the adjusted findings also indicate that these conflict dimensions should be interpreted cautiously as correlates of turnover rather than as independent determinants of actual job leaving.

One of the important strengths of our study is that it addressed not turnover intention, but actual turnover behavior observed at the end of the six-month follow-up period. Much of the literature has relied on turnover intention as an outcome. For example, Saigusa et al. (19), in their cross-sectional study conducted with hospital nurses in Japan, showed that work–family conflict was associated with turnover intention, whereas work engagement operated as a protective factor within this relationship. Similarly, the meta-analysis by Park and Min in the hospitality sector demonstrated that role stressors and work–family and inter-role conflict had strong effects on turnover intention (20). In a study conducted in Türkiye, Baş reported the mediating role of work stress in the relationship between work engagement and turnover intention (21). Our findings are consistent with this literature in direction; however, unlike many previous studies, the present study used employer-verified turnover data, thereby providing evidence at the level of behavior rather than intention (19–21).

The finding that work engagement was higher among employees who remained in the organization represents one of the central findings of this study. In the parsimonious adjusted model, each one-unit increase in work engagement was associated with lower odds of turnover. This result suggests that work engagement may function as a motivational and psychosocial resource during the early employment period. Mazzetti et al. (22) showed in their meta-analysis that work engagement was strongly associated with job satisfaction and organizational commitment and inversely associated with turnover intention. Lyu and Fan (23) also reported, from a gender perspective, that work–family conflict could weaken work engagement. In a more recent longitudinal study, Kobayashi et al. (24) demonstrated that both the effect of work on family and the effect of family on work were associated with lower work engagement among employees. Our data are consistent with this line of evidence; as work–family conflict increased, work engagement weakened, whereas higher work engagement was associated with a greater likelihood of remaining in the organization (22–24).

The findings regarding marital status and parental status require a nuanced interpretation. In the univariate comparisons, married employees and those with children had higher work engagement scores, suggesting that family life may provide a sense of meaning, responsibility, and motivation that strengthens psychological attachment to work. This interpretation is consistent with Çemberci et al. (25), who reported significant relationships between work engagement, marital status, work experience, and having children among flexible workers in the post-pandemic period. However, marital status and parental status were not included in the final parsimonious regression model because the revised modeling strategy prioritized theoretical relevance, univariate association with turnover, and reduction of model complexity. Therefore, the higher work engagement observed among married employees and those with children should be interpreted as a group-level psychosocial difference rather than evidence that family status independently predicts retention or turnover. Future studies should examine whether marital status, parental responsibilities, shift characteristics, work schedule control, and work engagement interact in shaping early employment outcomes.

The female-dominated composition of the study sample also deserves specific consideration. Women constituted 76.6% of the participants, which is atypical for many automotive manufacturing settings and may offer a distinctive perspective on work–family dynamics in blue-collar industrial work. In contexts where traditional gender roles remain influential, women may experience a disproportionate burden of domestic responsibilities, caregiving expectations, and family-related emotional labor in addition to paid employment. Such responsibilities may intensify the perceived conflict between work and family roles, particularly in shift-based and performance-driven production environments. However, in the adjusted parsimonious model, work–family balance dimensions did not remain statistically significant after adjustment. This may indicate that their association with turnover partly overlaps with work engagement, job-entry motivation, and early adaptation processes. Since gender-role expectations, household labor, and caregiving burden were not directly measured in this study, this interpretation should be considered exploratory. Future studies in manufacturing settings should examine gender roles, domestic workload, caregiving responsibilities, and supervisor support more directly to better understand how work–family conflict affects retention among women blue-collar workers.

The absence of significant differences across shifts and departments suggests that the main factor explaining work–family conflict may not simply be the formal name of the shift or the label of the production unit. Kelly et al. (26) showed that changes in job design and control over time could influence work–family conflict. Likewise, the meta-analyses by French et al. (27) and Kossek et al. (28) demonstrated that work–family-specific social support plays a strong role in reducing conflict. Therefore, the absence of departmental differences in our study suggests that the key determinant may not be the department itself, but rather the predictability of work schedules, supervisor support, workload distribution, and organizational support structures (26–28).

It is also noteworthy that employees who had chosen their jobs for positive reasons showed lower work–family conflict and higher work engagement, and they had lower turnover in the univariate analysis. The quantitative review by Christian et al. (29) showed that work engagement is a strong psychological bond with work and is associated with task and contextual performance. In the meta-analytic review by Bauer et al. (30), newcomer adjustment was reported to be associated with job satisfaction, organizational commitment, and remaining in the organization through role clarity, self-efficacy, and social acceptance. The meta-analysis by Saks et al. (31) also demonstrated that institutionalized socialization tactics reduce intended turnover and strengthen adjustment. Similarly, in our study, the fact that employees who started their jobs for positive reasons had lower conflict and higher work engagement suggests that early job-entry motivation and person–job fit may be relevant to subsequent personnel mobility (29–31). However, negative job preference reasons were not statistically significant in the adjusted parsimonious model, suggesting that their effect may overlap with work engagement and early psychosocial adaptation.

The theoretical interpretation of these findings may be strengthened by considering them within the Job Demands–Resources model. The cross-national meta-analysis by Allen et al. (32) showed that domain demands are strong determinants of work–family conflict and that these relationships may vary across cultural contexts. Matthews et al. (33) reported that family-supportive supervisor behaviors promoted work engagement and subjective wellbeing. The JD-R model emphasizes that job demands are linked to strain and exhaustion, whereas job resources are linked to work engagement through motivational processes (8, 9). Within this framework, the negative effect of work on family may be interpreted as a demand-related component of the work–family interface, while work engagement may be interpreted as a motivational resource associated with remaining in the organization. Especially among newly hired blue-collar workers, family-supportive supervision, predictable scheduling, fair workload distribution, and early socialization practices may be important for strengthening engagement, supporting adaptation, and potentially reducing early turnover.

Finally, our focus on actual turnover behavior enhances the interpretive strength of the study. Brewer et al. (34) examined actual turnover longitudinally among early-career nurses and demonstrated the necessity of longitudinal models for explaining real turnover behavior. Similarly, our study contributes to this literature by focusing on actual personnel mobility rather than intention. Overall, the findings indicate that work–family conflict dimensions were associated with psychosocial functioning and showed univariate differences, whereas work engagement was associated with lower odds of turnover in the adjusted parsimonious model. In this respect, the results may inform human resources and occupational health practices aimed at strengthening engagement and supporting early adaptation among newly hired workers in the automotive supplier industry.

### Limitations and strengths

One of the major strengths of this study is that it followed newly hired employees prospectively and evaluated the outcome on the basis of actual turnover verified through company records rather than self-report. However, several limitations should be considered. First, the study was conducted in a single company; therefore, the generalizability of the findings to different subsectors, organizational cultures, and production settings is limited. This limitation is further reinforced by the demographic composition of the study group. Women constituted 76.6% of the participants, which is atypical for many automotive manufacturing settings and likely reflects the specific production type, workforce structure, and job allocation practices of the participating facility. Therefore, the findings should not be generalized directly to the broader automotive blue-collar workforce, particularly to male-dominated production environments. Second, the number of turnover events was limited, which constrained the complexity of the multivariable logistic regression model. For this reason, a parsimonious adjusted model was preferred over a broader full model including multiple sociodemographic covariates. This approach reduced the risk of overfitting and provided a globally significant model; however, it also limited the ability to examine the independent contribution of several demographic variables in the same model. Third, variation in baseline tenure should be considered when interpreting the findings. Although all participants were newly hired and met the predefined 0–6 month tenure criterion, workers assessed shortly after hiring and those assessed closer to 6 months may have been at different stages of organizational adaptation. Baseline tenure in months was included in the parsimonious adjusted model; however, this adjustment may not fully capture qualitative differences in role clarity, familiarity with the work environment, supervisor relationships, and adaptation to shift-based work.

Fourth, because the scales were based on self-report, they may be subject to common method bias and social desirability effects. Fifth, the six-month follow-up period limits the evaluation of longer-term career continuity. Nevertheless, measuring work–family balance and work engagement during the early employment period and relating these psychosocial factors to employer-verified turnover provides an important contribution to the limited number of prospective studies in manufacturing settings. Future multicenter studies with longer follow-up periods, larger and more diverse study populations, and multilevel designs may explain these relationships in greater detail by incorporating variables such as shift design, supervisor support, overtime, pay fairness, department–job fit, baseline tenure trajectories, and workforce gender composition into the model.

## Conclusion and recommendations

This study showed that work–family balance dimensions and work engagement are closely related to psychosocial functioning during the early employment period among newly hired blue-collar workers in the automotive supplier industry. Workers who left employment had higher scores for the negative effect of work on family, whereas those who remained employed had higher work engagement scores.

In the parsimonious adjusted logistic regression model, work engagement was significantly associated with lower odds of turnover. Although the negative effect of work on family and negative job preference reasons showed significant univariate associations with turnover, these variables did not remain statistically significant after adjustment. These findings suggest that work engagement may be an important psychosocial resource for supporting early retention, while work–family conflict and job-entry motivation remain relevant for understanding early adaptation and workforce stability.

From an occupational health and human resources perspective, workplace strategies that strengthen engagement, improve supportive management practices, increase predictability in shift scheduling, facilitate early organizational socialization, and support work–family balance may contribute to more stable workforce retention in manufacturing settings.

Future studies conducted in different sectors, with larger and more diverse study populations and longer longitudinal designs, are likely to provide a more comprehensive understanding of the direction and strength of these relationships. In addition, qualitative research may contribute to a deeper understanding of the individual and organizational factors underlying early turnover decisions.

## Data Availability

The raw data supporting the conclusions of this article will be made available by the authors, without undue reservation.
